# A Deep Learning Approach to Position Estimation from Channel Impulse Responses †

**DOI:** 10.3390/s19051064

**Published:** 2019-03-02

**Authors:** Arne Niitsoo, Thorsten Edelhäußer, Ernst Eberlein, Niels Hadaschik, Christopher Mutschler

**Affiliations:** 1 Machine Learning and Information Fusion Group, Precise Positioning and Analytics Department, Fraunhofer Institute for Integrated Circuits IIS, Nordostpark 84, 90411 Nürnberg, Germany; thorsten.edelhaeusser@iis.fraunhofer.de (T.E.); ernst.eberlein@iis-extern.fraunhofer.de (E.E.); niels.hadaschik@iis.fraunhofer.de (N.H.); 2 Machine Learning and Data Analytics Lab, Friedrich-Alexander University Erlangen-Nürnberg (FAU), Carl-Thiersch-Straße 2b, 91052 Erlangen, Germany

**Keywords:** radio-based real-time locating systems, time difference of arrival, channel impulse response, time of arrival, position estimation, machine learning, deep learning, convolutional neural networks, distributed CNN

## Abstract

Radio-based locating systems allow for a robust and continuous tracking in industrial environments and are a key enabler for the digitalization of processes in many areas such as production, manufacturing, and warehouse management. Time difference of arrival (TDoA) systems estimate the time-of-flight (ToF) of radio burst signals with a set of synchronized antennas from which they trilaterate accurate position estimates of mobile tags. However, in industrial environments where multipath propagation is predominant it is difficult to extract the correct ToF of the signal. This article shows how deep learning (DL) can be used to estimate the position of mobile objects directly from the raw channel impulse responses (CIR) extracted at the receivers. Our experiments show that our DL-based position estimation not only works well under harsh multipath propagation but also outperforms state-of-the-art approaches in line-of-sight situations.

## 1. Introduction

Radio-based real-time locating systems (RLTSs) are key to drive automation and digitalization in many applications in warehouse management, production, and manufacturing. In contrast to highly accurate vision-based tracking systems, which often also raise privacy concerns and are prone to dirt and weather conditions, they guarantee a robust position tracking traded for a lower accuracy. As localization is also discussed for standardization in 5G, we can expect to see RF-based tracking becoming more and more ubiquitous especially in GPS-denied areas such as indoor environments [1].

Under the hood, RF-based positioning systems may be implemented using a multitude of different technologies, which include angle of arrival (AoA), received signal strength (RSS), time of arrival (ToA), and time difference of arrival (TDoA). While RSS- and AoA-based localization usually come at a low cost but also lower accuracy, T(D)oA-based systems require synchronization schemes and have a more complex system setup, which usually makes them more expensive. Nevertheless, their better positioning accuracy makes them still attractive for many use-cases, including the tracking of goods in warehouses or virtual and augmented reality in various applications [2,3,4].

However, achieving the application requirements through TDoA-based positioning may also become difficult in practice. The omnipresence of metallic surfaces, i.e., machinery, pipes, vehicles, racks etc., in industrial environments leads to effects such as signal reflections (multipath propagation), scattering, obstruction, shadowing, and attenuation [5]. These make the ToA-estimation non-linearly distorted, and if the effects are strong it increasingly becomes difficult to estimate the correct ToA from the radio burst signal. This leads to a erroneous position estimation. In practice, we often use much more infrastructure nodes than we would actually need for unambiguous position estimation (which results in an over-determined system) and try to install them at positions where those unwanted effects are minimal. This, however, requires a measurement campaign to capture the propagation effects on which we then need to solve a highly non-linear optimization problem.

Approaches that explicitly address multipath propagation include (unscented) Kalman filters [6,7], channel classification [8], subsample interpolation [9], sub-space approaches [10], or cone constraints [11,12]. But they only work well if there is still a strong line-of-sight (LoS) signal. Available methods that make explicitly use of multipath propagation, i.e., by generating scattering models with statistics [13,14], by simultaneous target and multipath positioning [15], by using training signals to model a random variable [16] together with a floor plan to enhance the tracking filter [17] or by using large-scale MIMO [18] do not scale well or do not improve with an increasing number of available training signals, which may often be acquired easily in practice.

This article builds on recent successes of deep learning (DL) [19] in order to estimate positions from raw channel impulse response data in a TDoA-based system setup and extends the work from [1]. In deep learning, we do not extract features (e.g., the ToA) from the signal manually. Instead, we allow DL to derive its own features from the raw data. We use ground truth position data generated by a robot equipped with an optical reference system to obtain reference labels for CIR samples and use them to train a deep convolutional neural network (CNN). The deep neural network models both the linear and multipath signal propagation effects in the environment and (once it has been trained) it can also be fine-tuned for other environments.

The remainder of this article is organized as follows. Section 2 discusses related work. Section 3 provides information on ToA estimation from channel impulse responses (CIRs) in RF localization systems and introduces the main concepts behind (convolutional) neural networks. Section 4 presents our approach to pre-process and normalize the raw channel impulse response data in order to apply convolutional neural networks on them. Section 5 describes our experimental setup and our datasets before we evaluate our position estimator on different real world datasets. Next, we present our experimental results in Section 6. We show that we outperform conventional position estimators not only in massive multipath environments but that we can also compete in line-of-sight scenarios. Section 7 concludes the article.

## 2. Related Work

There is much work that uses the RSS [20,21,22,23,24], the ToA [25,26,27], or their combinations [28]. Some use machine learning (ML) based schemes such as neural networks with a single hidden layer [21,26,28], variants of neural networks (i.e., deep belief networks [22,29], deep neural networks [30,31], fuzzy neural networks [32], artificial synaptic networks [25]), Gaussian regression [33], support vector machines (SVM) [27], random decision forest [34], or combinations of them [20]. Iqbal et al. [35] monitor patients using CNNs to correlate RSS measurement in a clinical environment. Ibrahim et al. [36] use CNNs and Sahar et al. [37] use long short-term memory (LSTM) recurrent neural networks (RNN) to process a time-series of RSS measurements. Feigl et al. [38] use LSTMs on ToAs in an TDoA system setup. Some recent work even estimates positions in a reinforcement learning setting [39]. However, all these methods manually extract features [40] along the RSS- or ToA-processing (or theirs combinations), which results in poor features for position estimation in a multipath environment.

A much richer feature to estimate the location of an object is the channel impulse response (CIR) [41]. Yu et al. [42] extract energy and delay features from the UWB impulse response and use these features to train a neural network. Hong et al. [43] model the multipath components (MPC) amplitudes in a UWB system using a Gaussian Process and lets it predict the CIR at arbitrary positions. A classification of line-of-sight and non line-of-sight (NLoS) situations using extracted features [44] has been proposed using SVMs [45] and Gaussian processes [46], while Choi et al. [47] yet use RNNs for a classification of NLoS over time for highly bandwidth-limited CIRs. There are also approaches that use deep learning on the CIR to identify NLoS situations [48]. Cui et al. [49] use a neural network to approximate the relationship between the SNR and statistical information such as skewness and kurtosis in the CIR. Savic et al. [50] propose a kernel-PCA combined with Gaussian process regression that projects the channel parameters onto a nonlinear space from which then a subset is used for ranging. Ergut et al. [51] use a set of anchors to generate multipath profiles, i.e., a number of time differences between peaks within a single CIR, which are used together with ground truth data to train a neural network with a single hidden layer. Jin et al. [52] approximate the CIR from subcarrier amplitudes of OFDM signals and propose a fingerprinting based on Gaussian regression. Also known as channel state information (CSI) this has extensively been studied lately [53]. However, all of the above approaches extract hand-crafted features from the CIR. Those only represent a subset of the available information, which only results in a rough estimation.

In contrast to a manual feature extraction, deep learning (DL) aims at finding and extracting the relevant features from the sensor data directly. This requires more data. Wang et al. [54,55,56,57] among others [58,59,60] propose several ideas to process the CSI from WiFi OFDM-signals, for instance, using deep CNNs. They feed the CSI directly into a CNN to estimate a position [54], train with phase information [55], directly estimate the angle of arrival with a CNN using phase fingerprinting [56], and also combine these ideas [57]. These ideas are similar to those that heavily make use of autoencoders for CSI-based fingerprinting [61,62,63]. However, their difference lies in the nature of the underlying signals and the system setup. TDoA-based localization requires a synchronized network of access points, i.e., anchors [1]. Different from ToA the subcarrier amplitudes describe the signal propagation profile and hence the relationship between access points and mobile devices at specific positions.

Tiemann et al. [64] use DL to estimate orientation-dependent error induction characteristics from the CIR. However, they do not consider CIRs from synchronized antennas and do not estimate the position. Vieira et al. [65] use convolutional neural networks to fingerprint massive MIMO channels, while others [66,67] use (deep) neural networks to estimate channel coefficients. However, the processed signals, the available information, and the system setup in massive MIMO channel fingerprinting are significantly different. Comiter et al. [68] propose a beam estimation for using deep neural networks that derives the angle of arrival by phase differences. Using different antenna arrays a structured pair of neural networks is used to estimate the antenna beam. However, although they use AoA measurements in a time-series manner to train a CNN for position estimation in [69] they do not estimate the position within a ToA-setup. Xiao et al. [70] propose denoising autoencoders to model the noise of reference locations. In the localization phase the measurement point is denoised by the autoencoder and a k-Nearest-Neighbor (KNN) classifier estimates the location. However, they train a DNN with raw RSSI measurements and then use the latent representation of the reference locations as fingerprints. Hence, both the setup and the signals significantly differ from our approach as we train the neural network end to end.

## 3. Background and Problem Formulation

### 3.1. Channel Estimation

Channel impulse responses (CIRs) are derived at each of the receiver units by the signals emitted by the mobile tags. We later use these CIRs for position estimation using a deep convolutional neural network (CNN). RTLSs that use time difference of arrival (TDoA) estimation need synchronized receivers. They are capable of estimating not only the position of the mobile tag but also its time-of-transmission (ToT). Both of them result from a hyperbolic trilateration of the TDoA values.

**Figure 1:**
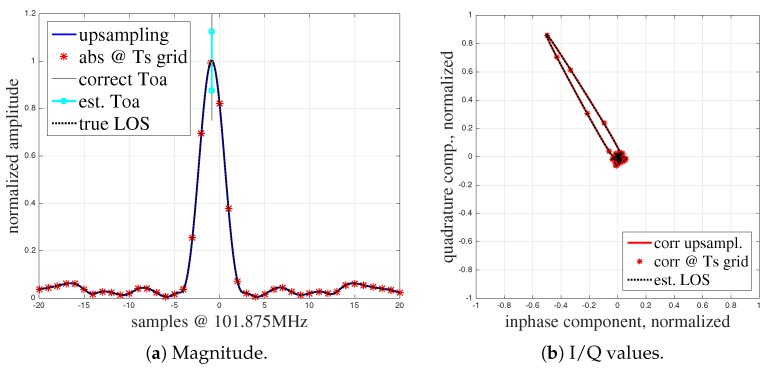
Characteristics of the correlation function.

We derive the ToA at each antenna through an analysis of the channel impulse response (CIR) that we obtain by transmitting e.g., pseudo-noise sequences and correlating them with the reference signal in the receiver. The ToA is then estimated by an evaluation of the correlation peak, see Figure 1.

Figure 1a shows the result of processing an ideal signal from the system (for details see Section 5) with a low frequency offset under LoS conditions. As the sampling frequency is limited, we need to interpolate the CIR (blue line) to obtain an accurate ToA estimation. The red stars mark the samples at the sampling frequency. Figure 1b shows the in-phase and quadrature components (I/Q). The I/Q diagram highlights the phase relationship of the I and Q components together with the magnitude (radius of correlator output in the complex plane). Without multipath propagation, the main information given in the I/Q diagram is the phase relationship between the carrier phase of the transmitted and the received signal.

We can extract the ToA by estimating the peak in the signal of Figure 1a (at this point the correlation between the measured received signal and the transmitted signal is at its maximum), which is approximately 19 ns from the start of the correlation window (see the vertical line). The correlation peak characteristics depend on the pulse shaping filter used for the generation of the bandwidth-limited signal and the sequence. The pulse shaping filter limits the used bandwidth of the signal. A typical peak width is in the range of 2 samples approx. at Nyquist sampling rate, i.e., with the sampling interval 1/B (bandwidth). Hence, although we process an almost undistorted CIR signal, we can only extract the real ToA with some variance that is introduced by the bandwidth-limited analog signal and noise within the wireless channel (in the LoS scenario). In order to find the real ToA from the CIRs an interpolation between sampling points is used together with inflection point ToA estimation [71], which searches for the point with the maximum gradient of the rising edge of the correlation curve (more details in Section 4.1). This, as well as averaging multiple ToA measurements (free running local oscillators) [6] may help to reduce ToA estimation errors to a minimum.

**Figure 2:**
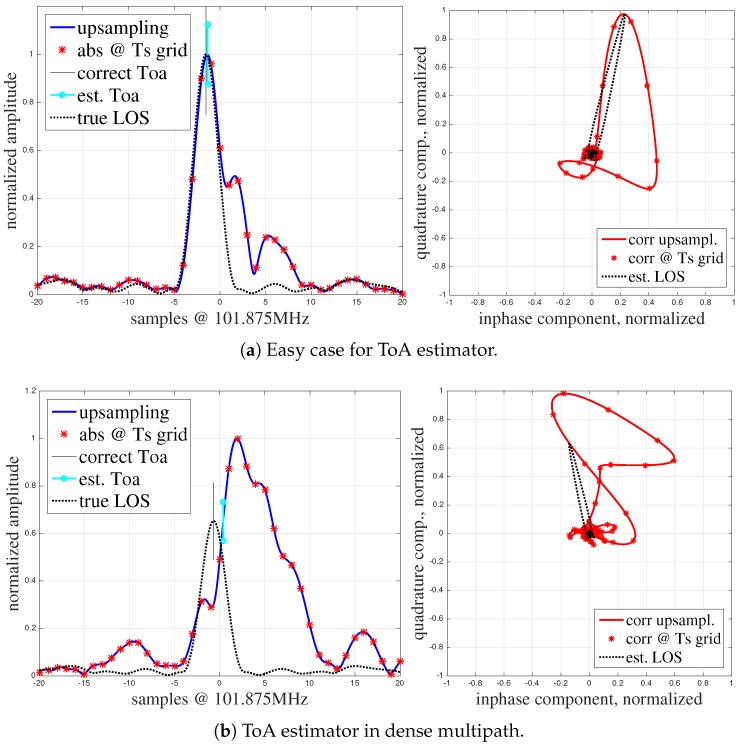
Examples for disturbed correlation functions. (**a**) A part of the signals arrive at a significant delay but the ToA estimator is still able to find the close-to-optimal ToA estimate. (**b**) ToA estimation in dense multipath (higher delay).

However, not only a limited sampling frequency introduces errors. Consider the red CIR in Figure 2b. Due to multipath propagation the signal travels along many paths until it reaches the antenna. Hence, in real environments, we need to take multipath propagation into account. Usually, the complex signal that arrives at the antennas is modeled as

(1) $$r\left(t\right)=\sum_{i=1}^{N_{m}}\alpha_{i}\left(t\right)e^{j\phi i(t)}\cdot s\left(t-\tau_{i}\left(t\right)\right)+n\left(t\right),$$

where s(t) is the transmitted single carrier signal sequence, $\alpha_{i}\left(t\right)e^{j\phi i(t)}$ and $\tau_{i}$ are the complex amplitudes and the tapped delay line, respectively, for each multipath component *i*. Additive white Gaussian noise n(t) is added on top of the received signal. The received signal is convolved with the same inverted conjugate complex transmit sequence to obtain the correlation $h\left(t\right)=r\left(t\right)*s^{*}\left(\tau -t\right)$. The multipath introduces additional superimposed correlation peaks. If the delay is significant, these peaks can be distinguished from the peak associated to the first arriving signal. This and the NLoS situation, i.e., the LoS signal is blocked and attenuated such that its strength is lower than the strength of the multipath signals, make the ToA estimation ambiguous. Figure 2 provides examples for such impaired correlation peaks. The figures include also the ToA estimated by state-of-the-art ToA estimators and the correct ToA. In case of multipath propagation, signals arrive with a small delay overlap, see Figure 2a. Depending on the magnitude and phase of the multipath components (MPC) different shapes of the first peak result in incorrect ToA estimates. In both Figure 2a,b the phase of the MPCs may be random.

There are a number of ToA estimators that make use of thresholds, maximum energy, and zero crossings to determine the correct ToA [49]. However, in multipath situations the true ToA can still only hardly be detected and cannot be estimated from the CIR alone without further information. Apparently, such ToA errors cause erroneous biased and noisy TDoAs, which may lead to a large bias in the position estimate. Thus, we investigate how to improve the precision in the LoS scenario and develop solutions for improving the accuracy in the heavily impaired NLoS scenario.

### 3.2. (Convolutional) Neural Networks

In order to put the position estimation in a formal context, assume that we are given many feature vectors $H\left(t\right)={\left[h_{0}\left(t\right),h_{1}\left(t\right),\cdots h_{i}\left(t\right)\right]}^{T}$ that hold the channel impulse responses $h_{i}\left(t\right)$ of *i* receivers and their labels, i.e., their respective ground truth positions, $p_{H(t)}={\left[t,x,y,z\right]}^{T}$ (time of transmission and Euclidean coordinates in three dimensions). Hence, we try to find a general function approximator θ that estimates a position from a previously unknown set of correlations H.

**Figure 3:**
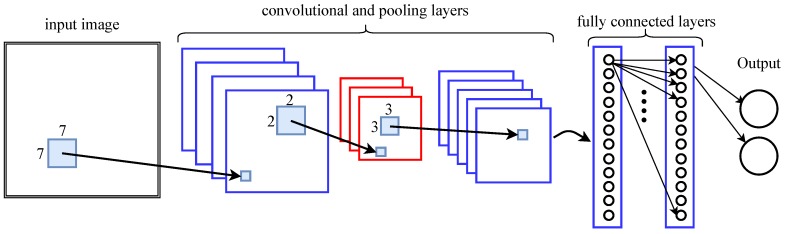
Principle architecture of a convolutional neural network [1].

Artificial neural networks consist of many interconnected simple units, i.e., neurons. Classic feed-forward networks have layers of fully connected neurons, as represented by the two concluding layers in Figure 3. The layers that we cannot see from the outside, i.e., any layers in between the input and the output layer, are called hidden layers. With data at the input layer the neurons propagate activations and provide information at the successive layer. Artificial neural networks are generalized function approximators and their depth (number of hidden layers) defines the complexity of the functions they can approximate. Hence, they may provide a suitable solution for the position approximation/estimation.

Each connection to a neuron has an assigned weight parameter *w* that controls the influence of the preceding neuron. A connection of the *i*-th neuron of layer *k* with the *j*-th neuron of layer k+1 is defined by a weight $w_{ij}^{k}$. In order to propagate the neuron activation throughout the network, we iteratively calculate the output $h_{j}^{k}\left(x\right)$ of any single neuron *j* per layer k>0

(2) $$h_{j}^{k}\left(x\right)=g\left(b_{j}^{k}+\sum_{i=0}^{n}w_{ij}^{k}x_{i}^{\left(k-1\right)}\right),$$

where $b_{j}^{k}$ is a bias parameter, $w_{ij}^{k}$ is the weight of the neuron connection, $x_{i}^{\left(k-1\right)}$ is the activation from the previous neuron, and g(·) is a (non-linear) activation function, such as a sigmoid function or the rectifier linear unit (ReLU). In practice, the weights of each layer are shared in a matrix and the network is evaluated using matrix multiplications along the layers.

Finding an optimal set of network parameters poses a non-convex optimization problem that is hard to be solved mathematically, i.e., it is NP-hard. But gradient-based optimization, such as stochastic gradient descent (SGD), works well in practice. Given a labeled data set (where both the input and the output labels are known), we can use the data as input, calculate the neuron activations layer by layer following Equation (2) and read the output of the network. On the output, we then apply a loss-function L, e.g., the mean-squared error, that defines how good the current network approximates the labels of the given data samples and that we want minimize:

(3) $$L\left(\theta ,H\left(t\right),{\vec{p}}_{H(t)}\right)=\frac{1}{2}{\left(\theta \left(H\left(t\right)\right)-{\vec{p}}_{H(t)}\right)}^{2}.$$

Next, we back-propagate the prediction error through the network, i.e., we calculate the influence of each neuron (activation) on the total error and use the gradients at each neuron as an indicator to reduce its influence on the error. Finally, we update the weight parameters using a (small) learning rate such that the loss decreases for future predictions.

After successful termination of the learning process, i.e., after some fixed number of iterations or by a threshold on the calculated loss, we determine the classification performance by applying test data to the neural network. If the performance values meet the desired criteria, the process of training a neural network is completed and it is ready to classify new data.

*Convolutional neural networks* (CNN) define a special architecture of neural networks. They use pooling layers and normalization layers interchangeably between consecutive convolutional layers, see Figure 3. The convolutional layers apply a convolution operation to the input (often the input is an image) to extract the features that are embedded in the training data. Each convolutional layer is also followed by a (non-linear) activation function. A convolution is a filter operator that is (conceptually) slid over the input and that preserves the spatial relationship between the input data points. Usually we apply several (different) of those convolutional filters on the same data in parallel. For instance, a 7 × 7 convolution filter next to 3 more run directly on the input image in Figure 3.

The pooling layers down-sample the data. Popular pooling operations are max- and average-pooling. The intuition behind them is that once the convolution layer has learned the features from the underlying data the pooling kernels (in Figure 3 with size of 3) run over the feature maps and keep the activations according to the pooling policy. This maintains the information while it reduces the spatial dimension and computation time.

Usually the first layers of a CNN model low level features such as edges and curves. As we stack more convolutional layers on top, the extracted features become more sophisticated. The fully connected layers at the end are used for classification (i.e., if we want to predict classes) or for regression (i.e., if we want to predict (real) numbers).

## 4. Data Preparation

### 4.1. Calibration of the CIR-s

**Figure 4:**
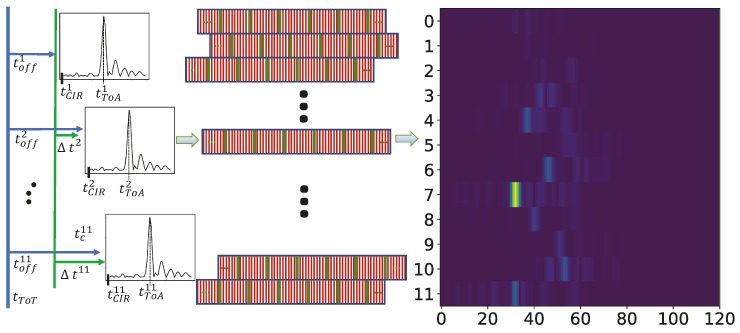
Data pre-processing: CIR calibration and padding.

Machine learning (ML) methods take training data to build up a model that can later be used to predict the value of a previously unseen data point. However in TDoA-based systems, the transmitter is not time-synchronized with the mutually synchronized receivers, i.e., the time-of-transmission $t_{ToT}$ is unknown. Hence, we also do not know at which point of time $t_{CIR}^{i}$ of a perceived channel impulse response at an antenna *i* actually starts. Often, a simple triggering method, e.g., a threshold method, is used to set the window of the estimated channel impulse response. However, this poses two challenges. Firstly, a single CIR only contains timing information relative to the window start time $t_{CIR}$ (delay of the LoS path relative to $t_{CIR}$ in Figure 4). Second, $t_{CIR}$ may not have a common timing across the receiving antenna channels *i*, due to varying analog signal processing delays, different lengths of antenna cables, and timing drift and jitter of internal clocks. Some of them are also affected by temperature changes. Hence, a set of CIRs originating from the same mobile tag position generally differs over time.

However, a simple calibration of timing offsets that normalizes $t_{CIR}^{i}$ helps to compensate for that. In order to do this, we derive a relative calibration offset $\Delta t^{i}$ per antenna unit *i* (with one antenna arbitrarily being set to 0) using reference transmitters at known locations (see the green time offset in Figure 4). We assume that we can install reference transmitters at positions with low multicast profiles, having LoS to the receiver antennas with low energy in multipath components. Then we derive the offsets by determining the ToA in reverse manner. As we know the positions of our reference transmitters and the receiver antennas, we know the correct ToA for the CIRs (of reference transmitters) and can derive the offsets for our receiver antennas. We use the calibration offsets to pre-process $t_{CIR}^{i}$ of the receivers such that all the CIRs together are approximately free of relative timing-offsets and hence stable over time. However, although this also adds a little bias to the position (as there is an error for the ToA estimates for the reference transmitters) it works fine in practice. This enables a TDoA-based estimation using the differences to a single antenna.

Now consider the CIR to be a time-discrete series of values that have been sampled with a specific sampling rate *f*, see the green lines in the middle of Figure 4. In radio-based locating systems the sampling rate is chosen to match the limitations of signal processing that inherit from the available bandwidth signal of the system. This also limits the accuracy of the resulting positions by design. For instance, the system we use for our experiments uses a sampling frequency of f=101.875MHz, which approximately results in a distance of c/f=c/101.875MHz≈2.94m (with *c* the vacuum speed of light) between two sampling points. However, an over-determined system (increasing the number of antennas) and interpolation between samples results in a reduced position estimation error.

As the resolution of the timing offsets $\Delta t^{i}$ is often more fine-grained than integral multiples of 1/f (see the green lines that represent the sampling points among the CIR windows in the middle of Figure 4), we have to re-adjust the discrete CIRs into the same timing units. We resample the CIR by a factor of n=100 (resulting in a distance of 2.94 cm between two samples) and use an anti-aliasing FIR low-pass filter with kaiser window that also compensates for the filter delay. Hence, we (1) interpolate the signal with a resampling factor *n* (see the red lines in Figure 4), (2) shift it sample-wise by the integral timing offsets $\Delta t^{i}/f\cdot n$ and (3) down-sample the signals by its resampling factor *n* again.

We further zero-pad the CIRs inside an N×M matrix with *N* antennas and *M* samples, that each CIR has the correct relative timing to each other and the median of all center of columns *m* is M/2. The median will compensate a small number of wrong detections of CIR, which can be caused by falsely detecting the CIR window start position. Samples not occupied by CIR values are padded with zeros. Now we have the CIRs calibrated and sampled at common timing units (such that the beginning of each window is aligned). We further interpret the matrix N×M×C as an image with its real and imaginary parts as different channels *C* as denoted on the right hand-side of Figure 4.

### 4.2. Normalization of Data

Usually, ML (and DL in particular) requires a normalization of the dataset. In the training of an ML model statistical properties (such as the mean and the standard deviation) are only computed from the training data, not from the validation or test data. Even if statistical properties are not explicitly computed the model converges in respect to the statistical properties of the underlying training data set. Hence, we must standardize the statistics for the validation and test data with the ones computed from the training data.

In deep learning we often *center* the data and hence remove statistical dependencies between training and test data. Normalization techniques are highly application- and data-dependent. In image classification we usually subtract the mean image of the training data from the test samples. We then see if the model captures statistical dependencies of the underlying data. Often we can think of it as a way to normalize the dataset such that it has a zero mean and standard deviation of 1.

Initial trials showed poor results for conventional normalization methods. The main reason is that the correlation signals are highly affected by non-linear effects. Hence, we do not subtract a *mean* correlation from the data but we normalize each input on its own. In previous experiments we investigated on several different scaling/normalization schemes, i.e., scaling per receiving antenna, scaling over the whole available data set, scaling over the preprocessed correlation image etc. It turns out that we obtain the best results if we consider the signals from *n* antennas units as a single set. We use a width of *m* = 60 to describe a correlation over time in its real and imaginary part, resulting in an n× 60 ×2 matrix. For each set we take the minimum and subtract that from each value, which represents the noise floor level of the CIR signal. We next divide by the maximum value to effectively scale the values to $\left[0;1\right]$. We apply this to real and imaginary signals separately.

Afterwards we combine the real and imaginary signals with their corresponding correlations values into a 2-channel correlation image of shape 12 × 120 × 2 (our system uses 12 receivers) that also considers recalibration and padding, see Figure 4 (right). These data samples are then free of any absolute time information. We need to keep an absolute time stamp for the correlation image (i.e., that of the statically chosen offset receiver) next to its reference position. Any other (relative) timing information is (implicitly) encoded in the matrix/image.

## 5. Experimental Setup

In order to validate our DL approach for position estimation, we record several measurement data sets using different setups. We describe our measurement infrastructure in Section 5.1, our datasets in Section 5.2, and our deep learning setup and model configuration in Section 5.3.

### 5.1. Measurement Infrastructure

The core of deep learning methods is a large dataset that is used to train, validate and test the model. In addition to the CIR data, for the training and the evaluation of our model we also need precise ground truth reference position data to label our training and test data sets. We obtain such labels with a Nikon iGPS system, i.e., an optical laser-based tracking system with a mean average error both vertically and horizontally below 1 mm and an update rate of 30 Hz.

**Figure 5:**
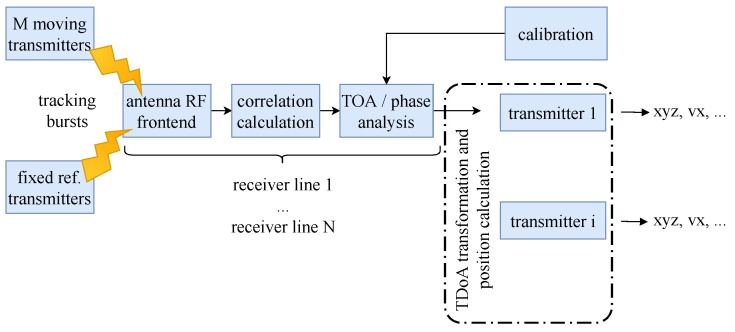
RedFIR’s signal processing chain.

For our experiments we also need a radio-based locating system that delivers a stream of channel impulse responses. We generated CIR data with a custom radio-based locating system that runs in the globally license-free ISM (industrial, scientific, and medical) band of 2.4 GHz and that uses around 80 MHz signal bandwidth [72]. Miniaturized transmitters use the available bandwidth to generate short broadband signal bursts together with identification sequences on which we correlate on the antenna units. Figure 5 illustrates the signal processing chain. The system distinguishes fixed reference transmitters for calibration purposes from *i* moving transmitters. All transmitters emit tracking burst signals, which are received by *N* receiving antennas. Our installation uses 12 antennas that receive signals from up to 144 different moving transmitters. Mobile tags emit up to 2000 tracking bursts per second (we use 200 bursts per second). The locating system allows to receive 50,000 of those signal bursts per second (per antenna). For each of the 12 receiver lines FPGAs correlate the burst sequences to obtain the correlation function that approximates the CIR. We ignore the ToA analysis and work directly on the CIR streams i.e., the output of the correlator. As the receivers are synchronized all the CIRs share a common time base.

We recorded the data in the Fraunhofer IIS L.I.N.K. (localization, identification, navigation, communication) test center in Nürnberg that provides a unique test ground on 1400 m^2^. We use the following platforms to collect our training data in order to capture different properties of tracking.

**Figure 6:**
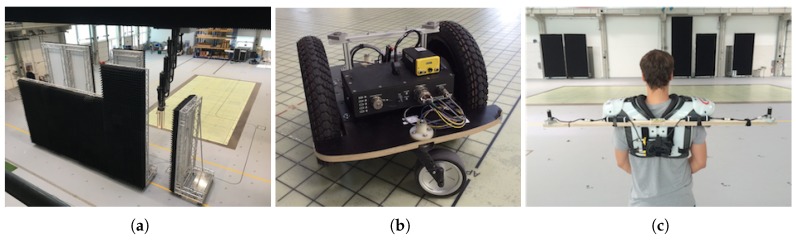
Platforms training data generation. (**a**) Positioning system [1]. (**b**) Segway. (**c**) Human.

**Positioning System.** We use a crane-like apparatus that approaches any 3D position in the area with repeating accuracy of <2 mm at a maximum speed of 3 m/s. Figure 6a shows the crane as it passes through a construction of absorber walls. We use the positioning system to capture a homogeneously distributed dataset that covers a larger area at a fixed height. The positioning system returns 25 positions per second, which however, are not as accurate as the iGPS position. Hence, we mounted not only the RF-tags but also the iGPS transmitters to the positioning system. As the system runs slowly we simply interpolate the reference positions (as the update rate of iGPS is lower than that of RLTS).

**Mobile Robot.** We use a Segway RMP-210, see Figure 6b, with a maximum speed of 30 km/h and an acceleration of 2 m/s^2^ to capture highly dynamic tracking data. As a reference we take the iGPS position and interpolate intermediate reference positions by interpolation and odometry information.

**Human.** We also use a body-mounted apparatus, see Figure 6c, that captures movements of persons. In general it allows to capture high velocity speeds, but usually the speed is below 10 km/h. We use several iGPS transmitters to determine the position of a mobile RF-tag that is located near the person’s neck [73].

### 5.2. Datasets

**Figure 7:**
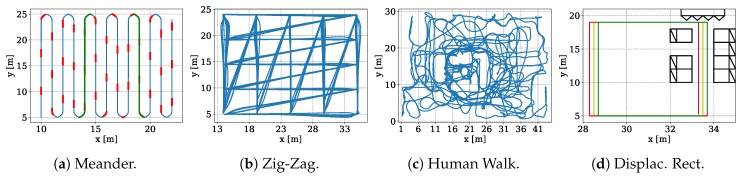
Datasets we use for our experiments [1]: Meander recorded with the positioning system; Zig-Zag recorded using the Segway RMP-210; Human Walk recorded using a human that builds up a construction in the test center; Displaced Rectangles recorded with the positioning system, see also Figure 6.

Figure 7 shows the measurement trajectories of our datasets. The platforms follow the trajectory and we record 200 correlation signals per antenna and second. As the iGPS system only delivers 30 Hz ground truth positions we interpolate both the ground truth positions and timestamps, see Section 5.1.

Table 1 specifies our datasets. From the total number of recorded training samples, we only select mostly complete sets (whenever we receive correlations from 11 of the 12 antennas). In a few cases correlations are corrupted, or low signal-to-noise ratios have been detected. We then discard such measurements. However, due to the heavy multipath in the displaced rectangles dataset (the rectangles used the path from Figure 6a) the system is only able to successfully correlate on 92,724 out of 218,752 burst signals to decode the CIRs. While we record 3D positions in any cases, we fixed the *z*-coordinate where possible as we only evaluate the 2D position accuracy as the sub-optimal geometry of the RF-antennas would a bias to our evaluation. As we could not fix the height of the transmitters for the random human walk we have varying heights embedded there.

### 5.3. Deep Learning Setup, Model Configuration and Data Processing

We ran all our experiments on a desktop machine equipped with an Intel Xeon E5-1620v4 CPU@3.5GHz (4 cores, 8 threads, 10 MB cache), 16 GB of main memory, and an NVIDIA GeForce GTX1080 GPU with 32 GB memory. We have implemented all our algorithms in C++14 on Ubuntu 16.04 LTS and use the caffe deep learning framework [74].

In pre-tests we have evaluated several different and well-known deep learning architectures such as AlexNet, VGG-16, VGG-19, and GoogLeNet [75]. It turned out that the GoogLeNet not only offers the best trade-off between depth of the network and number of parameters (and hence the training time), but also benefits from its inception modules. An inception module has a 1×1 convolution that reduces the dimensionality of a feature map. These are applied prior to computationally intense 5×5 and 3×3 convolutions, essentially giving multiple opinions of the same input data. The GoogLeNet is 22 layers deep, uses 9 inception modules, and has 2 intermediate classifiers. We made the following changes to the GoogLeNet architecture in order to facilitate the size of our correlation input:

- We replace each of the 3 softmax classifiers by affine regressors (Euclidean distance).
- We replace the fully connected (FC) layer that has 1000 output units (before the classifier) by an FC layer of 2 units outputting a vector of positions (x,y).
- We modify the max-pooling layer after the 2nd inception module to have a kernel size of 2 instead of 3, the avg-pooling layer at the first and second classifier to a kernel size of 3 instead of 5, and the max-pooling layer before final inception modules to a kernel size of 2 instead of 3.

For training we apply stochastic gradient descent (SGD) with a starting learning rate of $10^{-5}$ and an inverse decay, and applied a batch size of 50 for training and 10 for testing.

Activation functions play an import role in deep neural networks as they introduce nonlinearity to the mapping of the inputs to the outputs. The most commonly used activation functions include the sigmoid function ($f\left(x\right)=\frac{1}{1+e^{\beta x}}$), tanh ($f\left(x\right)=1-\frac{2}{1+e^{-2x}}$), and ReLU (f(x)=max(0,x)). Sigmoid and tanh both squish the activation to a value within $\left[0;1\right]$ and $\left[-1;1\right]$. For deep neural networks this causes the *vanishing gradient problem* [76], i.e., the gradient is close to zero at many points except around the middle, which makes training converge only slowly. In recent years the rectified linear units (ReLU) has become very popular. The function returns 0 whenever it receives negative input and with positive values it acts linearly and returns the value.

Considering that channel impulse responses may contain both positive and negative values, we might think that both tanh and sigmoid are suitable activation functions (as ReLU would just zero out negative correlation values). While initial experiments show that tanh yields slightly better results (however being very time-consuming in training) we obtained the best results if we only process positive correlations, i.e., we turn any negative number to a positive number. Using the ReLU activation function on negative input (either direct or indirect using negative weights) immediately causes the output of the neuron to become zero, which results in *dead neurons*. The gradient stays at zero and never changes again. Hence, we omit the DC-offset from the correlation and use ReLU. This yields a 28% better accuracy compared to a processing of both negative and positive correlations and a 20% better accuracy over tanh.

## 6. Results

**Figure 8:**
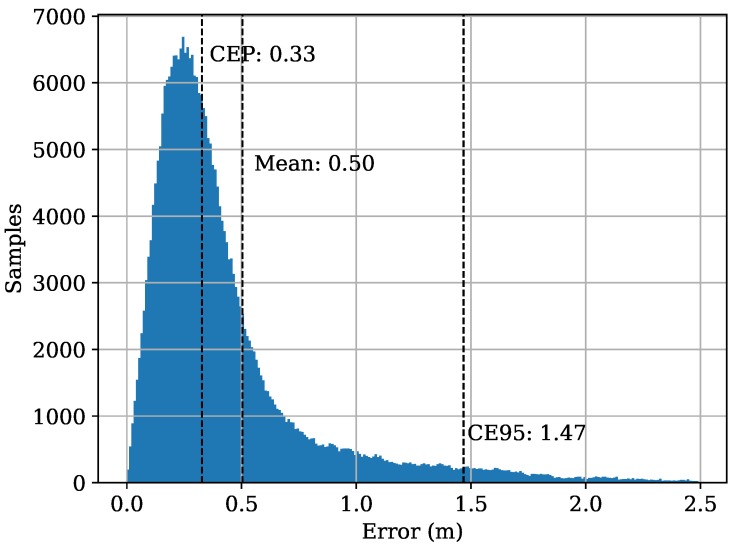
Distribution of error values after removal of outliers.

In order to get a baseline positioning performance of the RF-system we extract the ToAs under LoS conditions using the inflection point method [71] and run a Levenberg-Marquardt (LM) optimizer to obtain the positions. For each set of ToAs we run the optimization 12 times with the ToA of each receiver being once set to zero for one run of the LM and select the iteration with the lowest error term (best fit of TDoAs to position). On the Zig-Zag dataset we obtain an MAE of 0.50 m, a CEP of 0.33 m, and a CE95 of 1.47 m if we remove extreme outliers (>10 m) (we did not apply advanced outlier removal techniques such as Huber weighting [77], Chauvenet’s criterion [78] or Peirce’s criterion [79] but only a simple test of the resulting positioning error). Figure 8 shows the error distribution. In practice phase analysis or motion models improve accuracy.

### 6.1. General Performance Evaluation of the ML Approach.

For a simple performance experiment, we divide the datasets into training (80%) and testing (20%) set. We uniformly sample among the data points. Later we evaluate the model by the whole set and employ the Euclidean distance as a quality measure for the accuracy of the model.

Table 2 shows the results for our datasets employing DL. We achieve the best accuracy w.r.t. all metrics on the *Displaced Rectangles* (Figure 7d) and *Meander* (Figure 7a) datasets. However, the horizontal distance between the rectangles in *Displaced Rectangle* is only 0.2 m. We assume that the multipath components enrich the CIR information such that the model manages to estimate the position exceptionally good. The ZigZag (Figure 7b) hints that the speed of the Segway system has an effect on the CIR measurements. The accuracy is worst for the Human Walk Figure 7c dataset because the height of the mobile tag varies. The model has not obtained any information of the *z*-axis and hence cannot generalize the measurement on the xy-plane. As the CEP is smaller than the MAE, this means that we can easily filter out the outliers in a post-processing step. But on all our datasets (even on the Zig-Zag with high velocity and the LoS datasets) our DL-approach considerably outperforms the Levenberg-Marquardt optimization on the extracted ToAs.

### 6.2. Slicing Evaluation

Randomly sampling training and testing data does not elaborate whether the model generalized over the dataset or overfit to the training data. In order to check for generalization we construct two additional scenarios on the Meander dataset. *(1) Short-Slice (SS)* uses the correlations from the red slices in Figure 7a to test the model, while we use the rest to train the model. The test slices are approximately 1 m long. In this setup we evaluate small-scale generalization [80]. *(2) Long-Slice (LS)* uses the correlations from the green lines in Figure 7a to test the model while the rest is used for training. This evaluates large-scale generalization which helps us to see better how the model manages to approximate the position of the missing data points.

**Figure 9:**
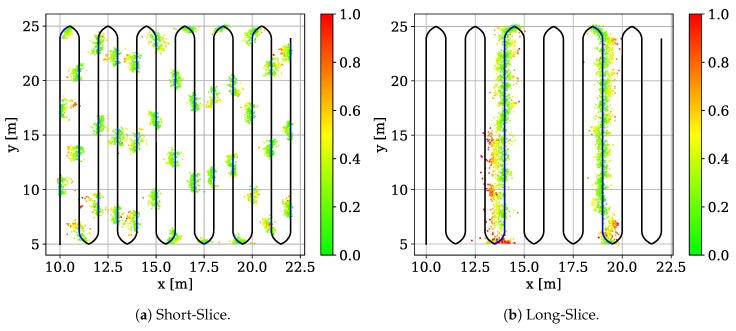
Evaluation of the model generalization [1].

Figure 9 shows color-coded error plots for SS (left) and LS (right). SS has an MAE of 0.27 m, a CEP of 0.24 m and CE95 of 0.57 m, while LS has an MAE of 0.34 m, a CEP of 0.27 m and a CE95 of 0.77 m. The precision of both tests degrade w.r.t. to the results that we achieve using the naive sampling approach (which however, did not check for generalization). But both results also show that our model generalizes over the training data set. Hence, without having seen a CIR data point in the direct neighborhood of the test data sample the model produces viable position estimates that are more accurate than the baseline optimization using the Levenberg-Marquardt algorithm.

### 6.3. Architecture Evaluation

**Figure 10:**
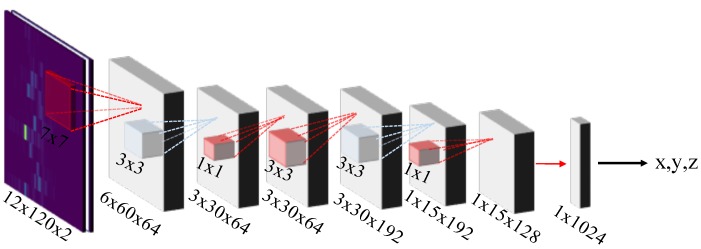
SmallNet architecture.

In order to determine the suitability of popular DL architectures, we modify AlexNet, GoogleLeNet, VGG-16 and VGG-19 according to Section 5.3 and train them with our correlation data. We further modified the GoogleNet, see Table 3. The *-Re* modification preserves the CIRs further down the network (we modified the initial convolutional and pooling layers). The *Re-NoP* preserves the size of correlation image (by removing the pooling layer between the first convolutional and normalization layer). We also defined *SmallNet*, see Figure 10, as a cut-off from the GoogLeNet architecture (we removed the inception layers between the root and the first intermediate output).

**Figure 11:**
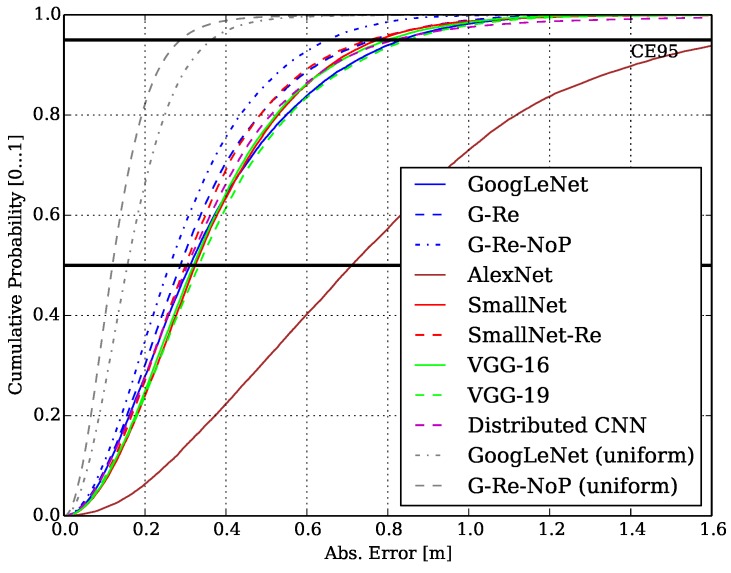
Cumulative Probability over the Meander dataset [1].

Figure 11 shows the CDFs of the network architecture trained and tested according to the LS scheme of Section 6.2. The graph also shows the CDF of the Meander dataset according to Section 6.1 with uniform sampling denoted as gray dotted line with the GoogLeNet and its modification G-Re-NoP as baseline for comparison. Table 3 specifies the evaluated architecture parameters together with the inference time per 1000 samples, MAE and CEP. GoogLeNet and VGG-19 are on par especially on the CE95-level. Surprisingly, VGG-16 outperforms VGG-19 with its modifications. AlexNet has the worst overall performance compared to the other network architectures (as it is comparably shallow but mostly fully connected throughout the network).

The G-Re-Nop provides the best shaped CDF and MAE but however, its inference time is considerably higher than that of others and also not feasible for practical applications of RLTS. Comparing GoogLeNet and SmallNet, we can see that they are on par w.r.t. MAE and CEP. While SmallNet has nearly 4 times less parameters and a 6 times faster inference time. The modified SmallNet-Re has slightly better CDF as the SmallNet at the cost of a larger number of parameters (approx. 2 million vs. 11 million), which also results in higher inference times.

### 6.4. Data Preprocessing and Zero Padding

In Section 4.1 we have argued for our calibration and zero padding strategy that aligns the correlations within the input image and that implicitly encodes relative timing information. However, there is a reason to suspect that the neural network extracts most of the necessary information from the zeroes that are embedded in each line and that (especially) small values around the correlation peak and the peak itself do not contribute much to the position estimation at all.

**Figure 12:**
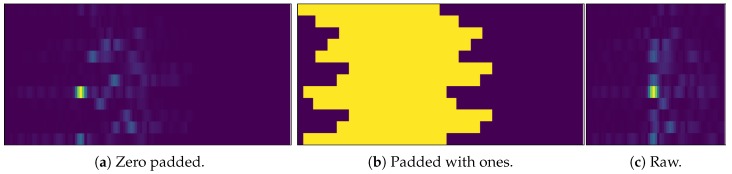
Overview: From raw data to zero padded to padded with ones.

In order to investigate the effectiveness of our preprocessing scheme we run two experiments, both using the LS scheme and dataset that we used in Section 6.2. (1) We generated a zero-padded correlation image of size 12 × 120 × 2 (Figure 12a) and replace the entries of the channel impulse response with ones. This results in the image depicted in Figure 12b. By that we get an impression how good the neural networks estimates positions from the rough timing information provided by the receiver synchronization (and in turn how much information it actually extracts from the CIRs when we compare the results). (2) We give up our zero-padding and directly stack the CIRs. This gives the smaller image of size 12 × 60 × 2 depicted in Figure 12c.

On the image from Figure 12a (the CIRs are both padded and present) the GoogLeNet achieves an MAE of 0.34 m, a CEP of 0.27 m and a CE95 of 0.77 m (see Section 6.2). Removing the CIRs from the correlation image (Figure 12b) lets it perform considerably worse, resulting in an MAE of 0.96 m, a CEP of 0.91 m and a CE95 of 1.89 m. If we only stack raw CIRs together (Figure 12c) we yield an MAE of 1.09 m, a CEP of 1.02 m and a CE95 of 2.18 m.

These results let us draw a number of conclusions. First, the CNN achieves a significantly worse performance if we process the CIRs according to Figure 12b. This is expected as the relative timing information that we used to align the CIRs within the correlation image only serves as a rough estimate of the transmitter’s position. In turn this means that the CNN heavily makes use of available CIR data that is embedded in the correlation image of Figure 12a. Second, the combination of the CIRs without padding from Figure 12c also performs significantly worse. But this effect is not as bad as we would have expected. Depending on the position of the mobile transmitter in the environment there might be a significant offset $\Delta t^{i}$ between the CIRs arriving at the receiving antennas and this has a big effect on position estimation. However, using the image of Figure 12c essentially turns out to be similar to CSI-based fingerprinting (where WiFi access points emit OFDM signals) [55]. The amplitude and the characteristics of signals emitted from mobile transmitters at a certain location help a lot to estimate (surprisingly accurate) positions.

### 6.5. Distributed CNN

In the previous experiments we focused on neural networks that process the CIRs in a central unit that provides enough computational resources. However, it not only takes high data rate communication links to transmit the CIRs. The central unit may easily become a bottleneck as it collects and preprocesses all the CIRs before it evaluates them with the neural network.

**Figure 13:**
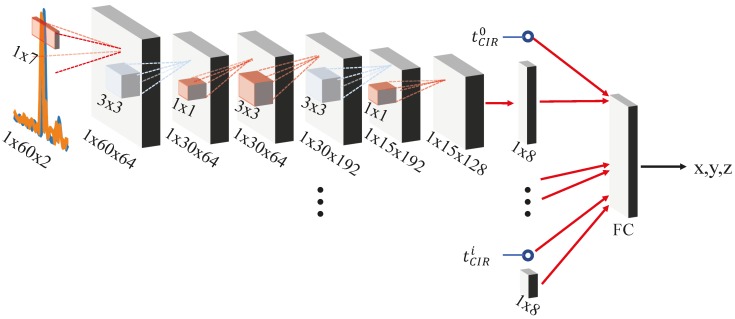
Distributed CNN.

Hence, we designed a CNN architecture according to Figure 13. The signals are preprocessed and a small CNN located near the receiver compresses the CIR by extracting a latent feature representation that only uses 8 neurons. For the preprocessing (1) we do not pad the signals but we recalibrate them according to their $\Delta t^{i}$, (2) we do not shift and resample the signals due to their CIR offset $t_{CIR}^{i}$ but we piggy-back $t_{CIR}^{i}$ to its latent feature representation, and (3) we do not scale the signals w.r.t. the values of the correlation image (as it is not available to a single receiver) but we normalize each CIR on its own given its mean and variance. Conceptually, we then transmit the activation of the 8 neurons and $t_{CIR}^{i}$ to the central computation unit. There the neuron activations and $t_{CIR}^{i}$ of all receivers are concatenated and processed in a fully connected layer that outputs the position. As before we train the model in an end-to-end way, i.e., we design the whole model for training. The individual parts can later be cut out from the whole network and deployed at the receiver units separately.

We trained and tested our model according to Section 6.2 and use the LS scheme to evaluate its performance. The distributed CNN architecture and preprocessing achieved an MAE of 0.36 m, a CEP of 0.30 m and CE95 of 0.82 m, see also Figure 11. This is remarkable also in comparison to the other (centralized) architectures. The distributed CNN manages to reach similar position performance although it processes information concurrently, only resolving inter-correlation information at a later point of processing, i.e., in the FC layer. It compresses the correlation signals at the receiver unit from size of 60 × 2 to 8 + 1 and hence achieves ∼13× compression, i.e., bandwidth requirement reduction, while it still maintains a high positioning accuracy.

### 6.6. Multipath Scenario

In reality there are many situations where the signal is attenuated, blocked or deteriorated. Real world environments often include scattering objects and obstacles that cause multipath propagation and blockage of the LoS signal. In order to see if our approach also manages to mitigate the effect of multipath we recorded the *Displaced Rectangles* dataset.

The rectangles in Figure 7d illustrate the trajectory. We use two of the three (red/left, green/right) rectangles for training and the middle one (yellow) for testing. Figure 6 shows how we placed absorber walls on the right side of the dataset. The perpendicular part on the rectangles’ right side heavily suffers from multipath propagation and obstructions. We ended up with a dataset of 92,724 CIR inputs and divided them such that the training set consisted of 62,724 and testing set of 30,000 correlations.

**Figure 14:**
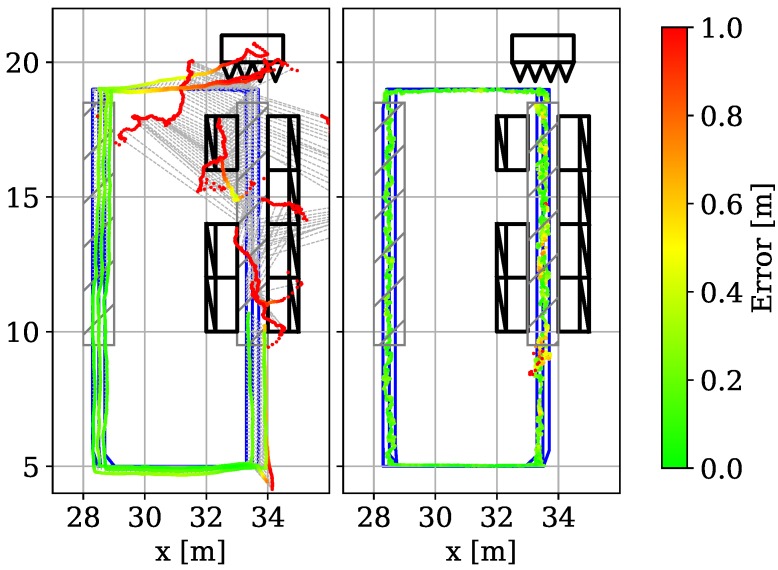
Results with multipath scenario [1].

Figure 14 on the left shows the color-coded result of state-of-the-art (SoTA) extended Kalman filter using a constant acceleration motion model that uses ToAs and phase information as input. Figure 14 on the right shows the results of our method. We use the yellow/middle trajectory for testing and a median filter for post-processing. We observe that classic ToA estimation and Kalman post-processing heavily suffers from NLoS situations. While ToA estimation together with the transition matrices of the filter perform very well on the left side (an MAE of 0.15 m, a CEP of 0.14 m, CE95 0.27 m in gray rectangle left) the highly non-linear effects on the right side cannot be resolved (MAE 2.11 m, CEP 1.45 m, CE95 5.29 m in gray rectangle right). Our approach is slightly worse in the LoS area with an MAE of 0.15 m, a CEP of 0.15 m and a CE95 of 0.28 m in the left rectangle due to the absence of a motion model. But most impressive is the NLoS performance: with a CEP of 0.23 m (MAE: 0.29 m, CE95: 0.68 m) our approach computes accurate positions even under heavy multipath (overall MAE: 0.17 m, CEP: 0.14 m, CE95: 0.45 m). We can hypothesize that the MPCs in the signals of the NLoS scenario can be viewed as virtual anchors or stations, which could be used for more precise position estimation [81,82,83]. This experiment shows that our approach efficiently handles the NLoS scenario. However, one limitation is that our approach (as any fingerprinting-based localization techniques) is sensitive to dynamic objects in the environment (as these change the multicast profile). In the future, it is worth to consider scenarios with moving objects to see how they affect the position estimation accuracy and long-term stability of our model.

## 7. Conclusions

This article presents a position estimation based on deep learning methods that directly operates on the channel impulse responses of TDoA-based locating systems. We provide details of our signal and data preprocessing and show the efficiency of our approach in different real world setups. While our approach keeps up with conventional signal processing approaches under line-of-sight conditions it outperforms previous approaches under heavy multipath propagation. The integration of a movement model may provide a boost in performance for the DL-based solution. We also introduced a concept that distributes the CNN so that it can be implemented in RTLS architectures.

The result of our research also lets us rethink how we currently estimate ToAs and that they may also be estimated by ML or DL approaches. This together with a consideration of historical measurements in a time-series may help to improve ToA estimation significantly. Next to this we plan to investigate the usage of simulation data for CNN pre-training so that we only require a little amount of actual training data in the target environment. Furthermore, in this work we did not yet consider the orientation of the transmitting explicitly although this also has an effect on the resulting CIR. We also did not discuss the effects to our calibration if our reference transmitters suffer from multipath propagation as well. However, as the estimates of those signals are only used for recalibrating the CIRs their influence to the final position accuracy of mobile tags is much lower (small error in the reference transmitter ToA-estimation level out over all the receiver units). However, in future work we investigate a combined processing of the mobile transmitter’s CIR together with the CIRs from the reference transmitters.

## Figures and Tables

**Table 1:** Description of datasets [1].

Dataset	# Samples	Covered Area (w × h)	Height	Platform
Meander	200,390 (211,416)	13 m × 20 m	2.5 m	Positioning System
Zig-Zag	304,120 (349,025)	22 m × 19 m	0.29 m	Segway
Human Walk	404,687 (691,680)	45 m × 30 m	0.96 m–2.1 m	Human
Displaced Rectangles	92,724 (218,752)	5 m × 14 m	2.8 m	Positioning System

**Table 2:** Results for general performance [1].

Dataset	CEP	CE95	MAE
Meander	0.16 m	0.36 m	0.17 m
Zig-Zag	024 m	0.67 m	0.29 m
Human Walk	0.30 m	0.87 m	0.36 m
Displaced Rectangles	0.10 m	0.24 m	0.12 m

**Table 3:** Results and parameters of different architectures [1].

Model	# Params	Avg. FP (ms)	MAE (m)	CEP (m)	CE95 (m)
GoogLeNet	6,894,976	66.30	0.36	0.31	0.83
G-Re	7,422,336	130.68	0.33	0.28	0.76
G-Re-NoP	8,778,112	411.68	0.29	0.26	0.65
AlexNet	34,535,104	24.46	0.79	0.71	1.68
SmallNet	2,113,664	10.83	0.36	0.32	0.77
SmallNet-Re	11,938,944	37.70	0.34	0.30	0.75
VGG-16	39,883,904	158.24	0.36	0.32	0.80
VGG-19	45,192,320	197.18	0.38	0.33	0.85
Distributed CNN	1,975,136	120.19	0.36	0.30	0.84

## References

[1] Niitsoo A., Edelhäußer T., Mutschler C. Convolutional Neural Networks for Position Estimation in TDoA-Based Locating Systems. Proceedings of the 9th International Conference on Indoor Positioning and Indoor Navigation.

[2] Gradl S., Eskofier B.M., Eskofier D., Mutschler C., Otto S. Virtual and augmented reality in sports: An overview and acceptance study. Proceedings of the 2016 ACM International Conference on Pervasive and Ubiquitous Computing, UbiComp Adjunct 2016.

[3] Feigl T., Mutschler C., Philippsen M. Supervised Learning for Yaw Orientation Estimation. Proceedings of the 9th International Conference on Indoor Positioning and Indoor Navigation.

[4] Roth D., Kleinbeck C., Feigl T., Mutschler C., Latoschik M.E. Beyond Replication: Augmenting Social Behaviors in Multi-User Virtual Realities. Proceedings of the IEEE Conference on Virtual Reality and 3D User Interfaces.

[5] Ruiz A.R.J., Granja F.S. (2017). Comparing Ubisense, BeSpoon, and DecaWave UWB Location Systems: Indoor Performance Analysis. IEEE Trans. Instrum. Meas..

[6] Nowak T., Eidloth A. (2011). Dynamic Multipath Mitigation applying Unscented Kalman Filters in Local Positioning Systems. Int. J. Microw. Wirel. Technol..

[7] Zhang C., Bao X., Wei Q., Ma Q., Yang Y., Wang Q. A Kalman filter for UWB positioning in LOS/NLOS scenarios. Proceedings of the 4th International Conference on Ubiquitous Positioning, Indoor Navigation and Location Based Services.

[8] He J., Geng Y., Liu F., Xu C. (2014). CC-KF: Enhanced TOA Performance in Multipath and NLOS Indoor Extreme Environment. IEEE Sens. J..

[9] Exel R., Bigler T. ToA Ranging using Subsample Peak Estimation and Equalizer-based Multipath Reduction. Proceedings of the IEEE Wireless Communications and Networking Conference.

[10] Driusso M., Babich F., Knutti F., Sabathy M., Marshall C. Estimation and Tracking of LTE signals Time of Arrival in a Mobile Multipath Environment. Proceedings of the 9th International Symposium on Image and Signal Processing and Analysis.

[11] Jin B., Xu X., Zhang T. A Fast Location Algorithm Based on TDOA. Proceedings of the 4th International Conference on Control, Mechatronics and Automation.

[12] Jin B., Xu X., Zhang T. (2018). Robust Time-Difference-of-Arrival (TDOA) Localization Using Weighted Least Squares with Cone Tangent Plane Constraint. Sensors.

[13] Al-Jazzar S., Caffery J., You H.R. A Scattering Model based Approach to NLOS Mitigation in TOA Location Systems. Proceedings of the 55th IEEE Conference on Vehicular Technology.

[14] Al-Jazzar S., Caffery J. ML and Bayesian TOA location estimators for NLOS environments. Proceedings of the 56th IEEE Conference on Vehicular Technology.

[15] Li L., Krolik J.L. (2014). Simultaneous Target and Multipath Positioning. IEEE J. Sel. Top. Signal Process..

[16] He J., Geng Y., Pahlavan K. (2014). Toward Accurate Human Tracking: Modeling Time-of-Arrival for Wireless Wearable Sensors in Multipath Environment. IEEE Sens. J..

[17] Meissner P., Leitinger E., Witrisal K. (2014). UWB for Robust Indoor Tracking: Weighting of Multipath Components for Efficient Estimation. IEEE Wirel. Commun. Lett..

[18] Garcia N., Wymeersch H., Larsson E.G., Haimovich A.M., Coulon M. (2017). Direct Localization for Massive MIMO. IEEE Trans. Signal Process..

[19] Kendall A., Grimes M., Cipolla R. PoseNet: A Convolutional Network for Real-Time 6-DOF Camera Relocalization. Proceedings of the 2015 International Conference on Computer Vision.

[20] Mascharka D., Manley E. LIPS: Learning Based Indoor Positioning System using mobile phone-based sensors. Proceedings of the 13th IEEE Annual Consumer Communications and Networking Conference.

[21] Martinez Sala A., Quir’os R., L’opez E. Using neural networks and Active RFID for indoor location services. Proceedings of the European Workshop Smart Objects: Systems, Technologies and Applications.

[22] Luo J., Gao H. (2016). Deep Belief Networks for Fingerprinting Indoor Localization Using Ultrawideband Technology. Int. J. Distrib. Sens. Netw..

[23] Prasad K.N.R.S.V., Hossain E., Bhargava V.K. (2018). Machine Learning Methods for RSS-Based User Positioning in Distributed Massive MIMO. IEEE Trans. Wirel. Commun..

[24] Prasad K.N.R.S.V., Hossain E., Bhargava V.K., Mallick S. (2018). Analytical Approximation-Based Machine Learning Methods for User Positioning in Distributed Massive MIMO. IEEE Access.

[25] Vaghefi S.Y.M., Vaghefi R.M. A Novel Multilayer Network Model for TOA-Based Localization in Wireless Sensor Networks. Proceedings of the International Joint Conference on Neural Networks.

[26] Singh P., Agrawal S. TDOA Based Node Localization in WSN using Neural Networks. Proceedings of the International Conference on Communication Systems and Network Technologies.

[27] Lewandowski A., Köster V., Wietfeld C., Michaelis S. Support Vector Machines for Non-Linear Radio Fingerprint Recognition in Real-Life Industrial Environments. Proceedings of the International Conference on Technical Meeting.

[28] Chen C.S. (2012). Artificial Neural Network for Location Estimation in Wireless Communication Systems. Sensors.

[29] Le D.V., Meratnia N., Havinga P.J.M. Unsupervised Deep Feature Learning to Reduce the Collection of Fingerprints for Indoor Localization Using Deep Belief Networks. Proceedings of the 9th International Conference on Indoor Positioning and Indoor Navigation.

[30] Félix G., Siller M., Álvarez E.N. A Fingerprinting Indoor Localization Algorithm based Deep Learning. Proceedings of the 8th International Conference on Ubiquitous and Future Networks.

[31] Kim K.S., Lee S., Huang K. (2018). A Scalable Deep Neural Network Architecture for Multi-Building and Multi-Floor Indoor Localization based on Wi-Fi Fingerprinting. Big Data Anal..

[32] Kuo R., Tseng W., Tien F., Liao W. (2012). Application of an Artificial Immune System-based Fuzzy Neural Network to a RFID-based Positioning System. J. Comput. Ind. Eng..

[33] Savic V., Larsson E.G. Fingerprinting-Based Positioning in Distributed Massive MIMO Systems. Proceedings of the 82nd IEEE Conference on Vehicular Technology.

[34] Akram B.A., Akbar A.H., Shafiq O. (2018). HybLoc: Hybrid Indoor Wi-Fi Localization Using Soft Clustering-Based Random Decision Forest Ensembles. IEEE Access.

[35] Iqbal Z., Luo D., Henry P., Kazemifar S., Rozario T., Yan Y., Westover K., Lu W., Nguyen D., Long T. (2017). Accurate Real Time Localization Tracking in A Clinical Environment using Bluetooth Low Energy and Deep Learning. PLoS ONE.

[36] Ibrahim M., Torki M., ElNainay M. CNN based Indoor Localization using RSS Time-Series. Proceedings of the 2018 IEEE Symposium on Computers and Communications.

[37] Sahar A., Han D. An LSTM-based Indoor Positioning Method Using Wi-Fi Signals. Proceedings of the 2nd International Conference on Vision, Image and Signal Processing.

[38] Feigl T., Nowak T., Philippsen M., Edelhäußer T., Mutschler C. Recurrent Neural Networks on Drifting Time-of-Flight Measurements. Proceedings of the 9th International Conference on Indoor Positioning and Indoor Navigation.

[39] Mohammadi M., Al-Fuqaha A., Guizani M., Oh J.S. (2018). Semisupervised Deep Reinforcement Learning in Support of IoT and Smart City Services. IEEE Internet Things J..

[40] Ye X., Yin X., Cai X., Yuste A.P., Xu H. (2017). Neural-Network-Assisted UE Localization Using Radio-Channel Fingerprints in LTE Networks. IEEE Access.

[41] Lin Y., Tseng P., Chan Y., He J., Wu G. (2018). A Super-resolution-assisted Fingerprinting Method based on Channel Impulse Response Measurement for Indoor Positioning. IEEE Trans. Mob. Comput..

[42] Yu L., Laaraiedh M., Avrillon S., Uguen B. Fingerprinting localization based on neural networks and ultra-wideband signals. Proceedings of the IEEE International Symposium on Signal Processing and Information Technology.

[43] Hong A.N., Rath M., Kulmer J., Grebien S., Van K.N., Witrisal K. Gaussian Process Modeling of UWB Multipath Components. Proceedings of the 2018 IEEE 7th International Conference on Communications and Electronics.

[44] Marano S., Gifford W.M., Wymeersch H., Win M.Z. (2010). NLOS Identification and Mitigation for Localization based on UWB Experimental Data. IEEE J. Sel. Areas Commun..

[45] Li W., Zhang T., Zhang Q. Experimental researches on an UWB NLOS Identification Method based on Machine learning. Proceedings of the 15th IEEE International Conference on Communication Technology.

[46] De Reyna E.A., Dardari D., Closas P., Djuric P.M. Estimation of Spatial Fields of Nlos/Los Conditions for Improved Localization in Indoor Environments. Proceedings of the 2018 IEEE Statistical Signal Processing Workshop.

[47] Choi J., Lee W., Lee J., Lee J., Kim S. (2018). Deep Learning Based NLOS Identification With Commodity WLAN Devices. IEEE Trans. Veh. Technol..

[48] Bregar K., Mohorčič M. (2018). Improving Indoor Localization Using Convolutional Neural Networks on Computationally Restricted Devices. IEEE Access.

[49] Cui X., Zhang H., Gulliver T. (2012). Threshold Selection for Ultra-Wideband TOA Estimation based on Neural Networks. J. Netw..

[50] Savic V., Larsson E.G., Ferrer-Coll J., Stenumgaard P. (2016). Kernel Methods for Accurate UWB-Based Ranging With Reduced Complexity. IEEE Trans. Wirel. Commun..

[51] Ergüt S., Rao R., Dural O., Sahinoglu Z. Localization via TDOA in a UWB sensor network using Neural Networks. Proceedings of the International Conference on Communications.

[52] Jin Y., Soh W., Wong W. (2010). Indoor Localization with Channel Impulse Response based Fingerprint and Nonparametric Regression. IEEE Trans. Wirel. Commun..

[53] Ghourchian N., Allegue-Martinez M., Precup D. Real-Time Indoor Localization in Smart Homes Using Semi-Supervised Learning. Proceedings of the 31st AAAI Conference on Artificial Intelligence.

[54] Wang X., Gao L., Mao S., Pandey S. (2017). CSI-Based Fingerprinting for Indoor Localization: A Deep Learning Approach. IEEE Trans. Veh. Technol..

[55] Wang X., Gao L., Mao S. (2016). CSI Phase Fingerprinting for Indoor Localization With a Deep Learning Approach. IEEE Internet Things J..

[56] Wang X., Wang X., Mao S. CiFi: Deep Convolutional Neural Networks for Indoor Localization with 5 GHz Wi-Fi. Proceedings of the IEEE International Conference on Communications.

[57] Wang X., Gao L., Mao S. (2017). BiLoc: Bi-Modal Deep Learning for Indoor Localization With Commodity 5 GHz WiFi. IEEE Access.

[58] Berruet B., Baala O., Caminada A., Guillet V. DelFin: A Deep Learning Based CSI Fingerprinting Indoor Localization in IoT Context. Proceedings of the 9th International Conference on Indoor Positioning and Indoor Navigation.

[59] Shao W., Luo H., Zhao F., Ma Y., Zhao Z., Crivello A. (2018). Indoor Positioning based on Dingerprint-Image and Deep Learning. IEEE Access.

[60] Wang Y., Xiu C., Zhang X., Yang D. (2018). WiFi Indoor Localization with CSI Fingerprinting-Based Random Forest. Sensors.

[61] Wu G., Tseng P. A Deep Neural Network-Based Indoor Positioning Method using Channel State Information. Proceedings of the International Conference on Computing, Networking and Communications.

[62] Yazdanian P., Pourahmadi V. (2018). DeepPos: Deep Supervised Autoencoder Network for CSI Based Indoor Localization. arXiv.

[63] Khatab Z.E., Hajihoseini A., Ghorashi S.A. (2018). A Fingerprint Method for Indoor Localization Using Autoencoder Based Deep Extreme Learning Machine. IEEE Sens. Lett..

[64] Tiemann J., Pillmann J., Wietfeld C. Ultra-Wideband Antenna-Induced Error Prediction Using Deep Learning on Channel Response Data. Proceedings of the 85th Vehicular Technology Conference.

[65] Vieira J., Leitinger E., Sarajlic M., Li X., Tufvesson F. Deep Convolutional Neural Networks for Massive MIMO Fingerprint-Based Positioning. Proceedings of the 28th Annual International Symposium on Personal, Indoor, and Mobile Radio Communications.

[66] Decurninge A., Ordóñez L.G., Ferrand P., He G., Li B., Zhang W., Guillaud M. CSI-based Outdoor Localization for Massive MIMO: Experiments with a Learning Approach. Proceedings of the 15th International Symposium on Wireless Communication Systems.

[67] Arnold M., Dorner S., Cammerer S., Brink S.T. On Deep Learning-Based Massive MIMO Indoor User Localization. Proceedings of the 19th IEEE International Workshop on Signal Processing Advances in Wireless Communications.

[68] Comiter M.Z., Crouse M.B., Kung H.T. (2017). A Structured Deep Neural Network for Data Driven Localization in High Frequency Wireless Networks. Int. J. Comput. Netw. Commun..

[69] Comiter M.Z., Kung H. Localization Convolutional Neural Networks Using Angle of Arrival Images. Proceedings of the IEEE Global Communications Conference (GLOBECOM).

[70] Xiao C., Yang D., Chen Z., Tan G. (2017). 3-D BLE Indoor Localization Based on Denoising Autoencoder. IEEE Access.

[71] Guenter Hofmann M.B. (2009). Device and Method for Determining a Time of Arrival of a Receive Sequence. U.S. Patent.

[72] Mutschler C., Ziekow H., Jerzak Z. The DEBS 2013 Grand Challenge. Proceedings of the 7th ACM International Conference on Distributed Event-Based Systems.

[73] Feigl T., Mutschler C., Philippsen M. Human Compensation Strategies for Orientation Drifts. Proceedings of the 25th IEEE International Conference on Virtual Reality and 3D User Interfaces.

[74] Jia Y., Shelhamer E., Donahue J., Karayev S., Long J., Girshick R., Guadarrama S., Darrell T. Caffe: Convolutional Architecture for Fast Feature Embedding. Proceedings of the 22nd ACM International Conference on Multimedia.

[75] Szegedy C., Liu W., Jia Y., Sermanet P., Reed S.E., Anguelov D., Erhan D., Vanhoucke V., Rabinovich A. Going Deeper with Convolutions. Proceedings of the IEEE Conference on Computer Vision and Pattern Recognition.

[76] Krizhevsky A., Sutskever I., Hinton G.E. ImageNet Classification with Deep Convolutional Neural Networks. Proceedings of the 25th International Conference on Neural Information Processing Systems.

[77] Zoubir A.M., Koivunen V., Chakhchoukh Y., Muma M. (2012). Robust Estimation in Signal Processing: A Tutorial-Style Treatment of Fundamental Concepts. IEEE Signal Process. Mag..

[78] Taylor J.R. (1996). An Introduction to Error Analysis: The Study of Uncertainties in Physical Measurements.

[79] Ross S. (2003). Peirce’s criterion for the elimination of suspect experimental data. J. Eng. Technol..

[80] Löffler C., Riechel S., Fischer J., Mutschler C. Evaluation Criteria for Inside-Out Indoor Positioning Systems Based on Machine Learning. Proceedings of the 9th International Conference on Indoor Positioning and Indoor Navigation.

[81] Ma Y., Wang B., Pei S., Zhang Y., Zhang S., Yu J. (2018). An Indoor Localization Method Based on AOA and PDOA Using Virtual Stations in Multipath and NLOS Environments for Passive UHF RFID. IEEE Access.

[82] Ulmschneider M., Luz D.C., Gentner C. Exchanging transmitter maps in multipath assisted positioning. Proceedings of the IEEE/ION Position, Location and Navigation Symposium (PLANS).

[83] Aditya S., Mloisch A.F., Behairy H.M. (2018). A Survey on the Impact of Multipath on Wideband Time-of-Arrival Based Localization. Proc. IEEE.

